# The diagnostic accuracy of clinical and laboratory parameters in the diagnosis of acute appendicitis in the adult emergency department population - a case control pilot study

**DOI:** 10.11613/BM.2018.030712

**Published:** 2018-10-15

**Authors:** Ivo Soldo, Vanja Radisic Biljak, Branko Bakula, Maja Bakula, Ana-Maria Simundic

**Affiliations:** 1 Surgery Clinic, University Hospital „Sveti Duh“, Zagreb, Croatia; 2 Department of Medical Laboratory Diagnostics, University Hospital „Sveti Duh”, Zagreb, Croatia; 3 Department of Ophthalmology, University Hospital Centre Zagreb, Zagreb, Croatia

**Keywords:** appendicitis, emergency service, white blood cell count, C-reactive protein, urinalysis

## Abstract

**Introduction:**

The evaluation of patients with suspected appendicitis strives to identify all patients with presenting symptoms while minimizing negative appendectomy rate. The aim of the study was to identify the optimal combination of clinical and laboratory parameters that should facilitate the emergency department surgeon’s definite decision.

**Materials and methods:**

The study group comprised 120 patients with suspicion of acute appendicitis (AA). In 60 patients the AA diagnosis was confirmed intraoperatively and by histological analysis. Clinical parameters included: appetite, vomiting, diarrhea, dysuria, signs of localized peritonitis and pain migration. Measured laboratory parameters were: C-reactive protein (CRP), complete blood count (CBC) and the urine test strip.

**Results:**

The control group of patients were more likely to present following symptoms: no changes in appetite (P < 0.001), diarrhea (P = 0.009) and dysuria (P = 0.047). CRP and white blood cell count (WBC) were significantly higher in the group with confirmed AA compared to the control group (44.7 *vs*. 6.6, and 13.6 ± 3.9 *vs*. 9.0 ± 3.4, respectively; P < 0.001). The multivariate logistic regression analysis identified lack of appetite (P = 0.013), absence of diarrhea (P = 0.004), and positive finding of signs of localized peritonitis (P = 0.013), as well as WBCs (P < 0.001) and negative urine test strip results (P = 0.009) as statistically significant predictors of AA. The highest percentage of correctly classified cases (82%) was achieved by combination of common clinical exam and basic inexpensive laboratory parameters (WBCs and urine test strip).

**Conclusions:**

Acute appendicitis in the emergency setting may be successfully ruled in based on elevated WBCs and negative urine test strip in combination with signs of localized peritonitis, lack of appetite and absence of diarrhea. Since CRP did not contribute to the overall diagnostic accuracy, its use in AA diagnostic protocols is of no value.

## Introduction

The lifetime risk of acute appendicitis (AA) in the general population is about 7% (*1*). Despite improvements in diagnostics and increasing surgical experience, the rate of negative appendectomies, even in most advanced medical centers, fails to fall below 10% (*2*). According to various studies the rates of false positive findings in the diagnosis of appendicitis vary from 8 to even 30% (*3*-*5*). Keeping in mind the possible serious and potentially fatal complications of unrecognized appendicitis, a relatively high rate of negative appendectomies (10-15%) is being considered acceptable among surgeons (*6*).

Diagnosis of AA is one of the most common dilemmas which surgeons encounter in the emergency room. The typical clinical picture, with pain migration towards the right lower quadrant of the abdomen or signs of localized peritonitis, is unfortunately found in much less patients than it is thought (*7*). On the other hand, too much reliance on laboratory findings can misguide a surgeon’s diagnosis. Broad differential diagnoses, especially in women, and an unclear clinical picture can frustrate a surgeon, leading him to order a Multislice Computed Tomography (MSCT) (*8*). However, although MSCT of the abdomen very accurately recognizes AA, it uses a high dosage of harmful radiation which makes it absolutely unacceptable for routine use in patients suspected of having appendicitis (*9*).

Searching through medical literature we can find various laboratory parameters (*e.g.* white blood cell count (WBC), neutrophil-to-lymphocyte ratio (NLR), platelets (Plt), mean platelet volume (MPV), C-reactive protein (CRP), fibrinogen and even bilirubin) being evaluated as potential diagnostic markers for AA, but results according to different studies vary substantially (*10*-*12*). On the other hand, some large meta-analyses found that individual laboratory and clinical parameters alone have low or no predictive value in the diagnosis of AA, but combined their predictive value increases a lot (*7*, *13*-*15*).

Many scoring systems and diagnostic pathways have been developed to improve diagnostic accuracy of AA (*16* - *18*). One of the most widely used today is the „Alvarado score“. Constructed in 1986 this scoring system is based on six clinical and two laboratory parameters (Table 1) where leukocytosis and right iliac fossa tenderness are considered the most important factors and therefore assigned with two points (*19*).

**Table 1:** Alvarado score for diagnosis of acute appendicitis

**Alvarado score**		Point*
	Migratory right iliac fossa pain	1
**Symptoms**	Anorexia	1
	Nausea/vomiting	1
	Tenderness in right iliac fossa	2
**Signs**	Rebound tenderness	1
	Elevated temperature	1
**Laboratory findings**	Leukocytosis (WBC > 10 x10^9^/L)	2
	Shift to the lest of neutrophiles (> 70%)	1
**Total**		**10**
*For each of the present symptoms, signs or laboratory findings, an adequate number of points is assigned. A score of 5 or 6 is compatible with the diagnosis of acute appendicitis. A score of 7 or 8 indicates a probable appendicitis. A score of 9 and 10 indicates a very probable appendicitis (*19*). WBC - white blood cell count.		

According to EAES (The European Association of Endoscopic Surgery) guidelines and based on research of Ebelle *et al.*, the Alvarado score determines quite accurately the probability of a patient having appendicitis if used with revised cut-off values (< 4 low risk, 4 - 8 intermediate risk, ≥ 9 high risk) (*16*, *20*). Although most guidelines acknowledge the overlapping similarity of symptoms in AA and urinary tract infection, especially in women, the most recommended laboratory tests for AA diagnosis include WBC and CRP, but not urinalysis (*16*–*18*). On the other hand, urinalysis is recommended as an inevitable part of the general assessment of acute abdominal pain (*18*). Whether urinalysis may improve the diagnostic accuracy of AA in emergency setting, is still unknown.

Our hypothesis was that the addition of urine test strip analysis to clinical and laboratory parameters could improve the diagnostic accuracy of AA diagnosis.

The aim of this retrospective study was to assess the diagnostic accuracy of clinical and laboratory parameters in the diagnosis of AA in the adult emergency department population.

## Materials and methods

### Subjects

This study represents a case-control pilot study. We retrospectively analyzed 120 (46 males) patients admitted to the Emergency Department of the University Hospital „Sveti Duh“ between February 2016 and February 2017 with a suspicion of acute appendicitis. The data were retrospectively collected through Hospital information system (SPP 2.0, Zagreb). Every patient with acute onset of right lower quadrant abdominal pain and without history of appendectomy was considered as suspected of having AA. This procedure is in accordance with the basic surgical education that every patient with right iliac fossa pain and without history of appendectomy is suspected of having appendicitis until proven otherwise. In 60 patients the AA diagnosis was confirmed intraoperatively and by histological analysis (AA group). The other 60 patients were processed and discharged, without confirmation of AA (control group). All discharged patients were confirmed as negative through follow up examination that occurred during following 2 to 3 days. Patients with palpable mass in right lower abdominal quadrant and patients with chronic pain were not analyzed. Patients with chronic right iliac fossa pain (longer than one month) are not suspected of having appendicitis because long experience of surgical dealing with acute abdomen showed that acute appendicitis never presents with longstanding pain. Palpable right iliac fossa mass is a sign of organized inflammatory mass around inflamed organ that is formed as a result of patient`s defensive mechanism („walled off appendicitis“) and therefore is a contraindication for operation. It is treated conservatively with antibiotics and delays appendectomy (4-6 months). This sign of palpable mass is rare and when present the management is clear - conservative treatment. This is the reason why most of the studies that are dealing with diagnosis of appendicitis also exclude patients with this sign (*21*, *22*). The Hospital Ethical Committee granted the approval for the retrospective analysis of the study data.

### Methods

Clinical and laboratory parameters, relevant to AA diagnosis, were analyzed among the study participants. Clinical parameters included: appetite, vomiting, diarrhea, dysuria, signs of localized peritonitis (rebound tenderness/guarding) and pain migration. Measured laboratory parameters included: inflammatory markers (CRP measured by immunoturbidimetry on the Beckman Coulter AU680 analyzer (Beckman Coulter, Brea, USA)); parameters of the complete blood count (CBC) (WBC, red blood cell count (RBC), red cell distribution width (RDW), Plt and MPV measured on the Siemens Advia 2120i 6-diff automated hematology analyzer (Siemens, Enlargen, Germany)); the urine test strip (iChem Velocity Urine Chemistry Strips for *in vitro* use with the automated iChemVELOCITY System (Beckman Coulter, Brea, USA)). Positive findings of blood (≥ 0.3 mg/L) and/or leukocytes (≥ 25 WBC/µL) on the test strip were considered as „positive result “.

### Statistical analysis

Normality of distribution was tested with Kolmogorov-Smirnov test. Patients were divided into subgroups with confirmed or rejected diagnosis of AA. All clinical variables, except age, are given in absolute number and percentages. The clinical and demographic variables were compared with the comparison of proportions test. Age is given in median and range and tested with Mann-Whitney test between groups. Measured variables that followed the normal distribution are expressed as mean value and standard deviation, while variables that did not follow the normal distribution were presented as median and interquartile range (IQR). Depending on the normality of distribution, the difference between two groups was tested with independent samples t-test or Mann-Whitney test. The Receiver Operating Characteristic (ROC) analysis was performed to estimate the measures of diagnostic accuracy in discriminating between AA and control group. Sensitivity (with 95% Confidence Interval (CI)) and specificity (with 95% CI) were calculated for all laboratory parameters. Logistic regression analysis was performed to identify the significant predictors of AA, among clinical and laboratory parameters. Statistical analyses were performed using MedCalc Statistical Software version 16.2.0 (MedCalc Software bvba, Ostend, Belgium). P < 0.05 was defined as the threshold of significance.

## Results

Comparison of basic demographic characteristics of the study subjects is shown in Table 2. The statistical analysis revealed that the AA group was older than the control group (P = 0.020). The patients with rejected diagnosis were more likely to present including symptoms: no changes in appetite (P < 0.001), diarrhea (P = 0.009) and dysuria (P = 0.047), while localized peritonitis (P = 0.027) and pain migration (P < 0.001) were significantly more prevalent in patients from the AA group (Table 3).

**Table 2:** Comparison of basic demographic characteristics between patients with confirmed acute appendicitis and in the control group

	**Control group** **(N = 60)**	**AA group** **(N = 60)**	**P**
**Male, N (proportion)**	11 (0.18)	35 (0.58)	< 0.001
**Female, N (proportion)**	49 (0.82)	25 (0.42)	< 0.001
**Age, years**	28 (17 - 66)	34 (17 - 84)	0.020
AA - acute appendicitis. The results are presented as absolute numbers and proportions, except age which is given in median and range (min-max). P < 0.05 was considered statistically significant.			

**Table 3:** Comparison of clinical presentation in patients with confirmed acute appendicitis and in the control group

	**Control group** **(N = 60)**	**AA group** **(N = 60)**	**Se (%)** **(95% CI)**	**Sp (%)** **(95% CI)**	**P**
**Appetite “YES”, N (proportion)**	35 (0.58)	13 (0.22)	78.3 (65.8 - 87.9)	58.3 (44.9 - 70.9)	< 0.001
**Vomiting “YES”, N (proportion)**	14 (0.23)	14 (0.23)	23.3 (13.4 - 36.0)	76.7 (63.9 - 86.6)	0.829
**Diarrhea “YES”, N (proportion)**	17 (0.28)	5 (0.08)	91.7 (81.6 - 97.2)	28.3 (17.5 - 41.4)	0.009
**Dysuria “YES”, N (proportion)**	11 (0.18)	3 (0.05)	95.0 (86.1 - 98.9)	18.3 (9.5 - 30.4)	0.047
**Signs of localized peritonitis (rebound tenderness/guarding) “YES”, N (proportion)**	3 (0.05)	12 (0.20)	20.0 (10.8 - 32.3)	95.0 (86.1 - 98.9)	0.027
**Pain migration “YES”, N (proportion)**	6 (0.10)	24 (0.40)	40.0 (27.6 - 53.5)	90.0 (79.5 - 96.2)	< 0.001
The results are presented as absolute numbers and proportions. AA - acute appendicitis. Se - sensitivity. Sp - specificity. P < 0.05 was considered statistically significant.					

The laboratory parameters were compared between the control and AA subgroups (Table 4). C-reactive protein and WBC were significantly higher in the AA group (44.7 *vs.* 6.6, and 13.6 ± 3.9 *vs.* 9.0 ± 3.4, respectively; P < 0.001). There was no statistically significant difference in RBC count, RDW, platelet count or MPV, between groups. The percentage of positive findings of the urine test strip was tested with the comparison of proportions test between groups. The test revealed no statistically significant difference (P = 0.555).

**Table 4:** Comparison of laboratory parameters in patients with confirmed acute appendicitis and in the control group

	**Control group** **(N = 60)**	**AA group** **(N = 60)**	**Criterion**	**AUC** **(95% CI)**	**Se (%)** **(95% CI)**	**Sp (%)** **(95% CI)**	**P**
**CRP, mg/L**	6.6 (1.2 - 46.1)	44.7 (26.1 - 94.4)	> 9.7	0.78 (0.70 - 0.85)	93.3 (83.8 - 98.2)	58.3 (44.9 - 70.9)	< 0.001
**WBC, x10^9^/L**	9.0 ± 3.4	13.6 ± 3.9	> 10.0	0.83 (0.75 - 0.89)	84.8 (73.0 - 92.8)	70.0 (56.8 - 81.2)	< 0.001
**RBC, x10^12^/L**	4.55 ± 0.47	4.69 ± 0.45	> 4.37	0.60 (0.50 - 0.69)	80.0 (67.0 - 89.6)	47.2 (33.3 - 61.4)	0.110
**RDW, %**	12.9 (12.5 - 13.4)	13.0 (12.8 - 13.5)	> 12.7	0.56 (0.46 - 0.66)	75.9 (62.4 - 86.5)	39.6 (26.5 - 54.0)	0.274
**PLT, x10^9^/L**	235 ± 54	217 ± 48	≤ 208	0.59 (0.49 - 0.68)	45.5 (32.0 - 59.4)	71.7 (57.7 - 83.2)	0.065
**MPV, fL**	10.5 ± 0.8	10.7 ± 0.7	> 9.7	0.57 (0.47 - 0.67)	94.3 (84.3 - 98.8)	18.9 (9.4 - 32.0)	0.164
**Urine test strip positive, N (proportion)**	20 (0.33)	16 (0.27)	/	/	73.3 (60.3 - 83.9)	33.3 (21.7 - 46.7)	0.555
The results are presented as mean ± SD or median (interquartile range, IQR). AA - acute appendicitis. Criterion - this value corresponds to the point on the receiver operating characteristic (ROC) curve farthest from the diagonal line; the possible threshold value with the highest specificity/sensitivity. AUC - Area under the ROC curve. Se - sensitivity. Sp - specificity. CRP - C-reactive protein. WBC - white blood cell count. RBC - red blood cell count. RDW - red cell distribution width. PLT - platelets. MPV - mean platelet volume. P < 0.05 was considered statistically significant.							

To identify variables that contribute most to the diagnosis of acute appendicitis, the logistic regression analysis was performed (Table 5). The univariate logistic regression analysis included clinical symptoms and laboratory parameters as independent variables. The confirmed/rejected AA diagnosis was a dependent variable. The univariate logistic regression identified lack of appetite (P = 0.002), absence of diarrhea (P = 0.001), dysuria (P = 0.017) and positive finding of signs of localized peritonitis (P = 0.014) as statistically significant independent predictors of AA. Vomiting and pain migration did not have an impact on predicting the appendicitis diagnosis outcome. Regarding laboratory parameters, the univariate logistic regression identified WBCs (P < 0.001) and negative urine test strip results (P = 0.020) as statistically significant predictors of the AA. The rest of the laboratory parameters did not have an impact on predicting the appendicitis diagnosis. Finally, we combined the significant variables from the univariate regression analysis and performed the multivariate logistic regression analysis. The results identified lack of appetite (P = 0.013), absence of diarrhea (P = 0.004), and positive finding of signs of localized peritonitis (P = 0.013), as well as WBCs (P < 0.001) and negative urine test strip results (P = 0.009) as statistically significant predictors of AA. The combination of clinical and laboratory parameters improved the percent of cases correctly classified compared to clinical and laboratory parameters alone (82% and both 77%, respectively).

**Table 5:** Logistic regression analysis for the identification of the optimum combination of independent variables for acute appendicitis diagnosis

**Significant variable**	**Coefficient**	**P**	**Odds ratio (95% CI)**	**Correctly classified cases (%)**
**Univariate analysis (clinical variables)**				
Appetite “YES”	- 1.71900	< 0.001	0.18 (0.07 - 0.44)	**77**
Diarrhea “YES”	- 2.20111	0.001	0.11 (0.03 - 0.43)	
Dysuria “YES”	- 1.75136	0.017	0.17 (0.04 - 0.73)	
Signs of localized peritonitis (rebound tenderness/guarding) “YES”	1.92275	0.014	6.84 (1.47 - 31.82)	
**Univariate analysis (laboratory parameters)**				
WBC, x10^9^/L	0.42010	< 0.001	1.52 (1.28 - 1.81)	**77**
Urine test strip positive	- 1.36071	0.020	0.26 (0.08 - 0.81)	
**Multivariate analysis (clinical symptoms and laboratory parameters combined)**				
Appetite “YES”	- 1.29674	0.013	0.27 (0.10 - 0.77)	**82**
Diarrhea “YES”	- 2.46968	0.004	0.08 (0.02 - 0.46)	
Signs of localized peritonitis (rebound tenderness/guarding) “YES”	2.19839	0.013	9.01 (1.58 - 51.40)	
WBC, x10^9^/L	0.38148	< 0.001	1.46 (1.22 - 1.75)	
Urine test strip positive	- 1.59636	0.009	0.20 (0.06 - 0.68)	
WBC - white blood cell count.				

## Discussion

The main findings of our study confirmed improved diagnostic accuracy of combined clinical and laboratory parameters in the diagnosis of AA in the adult emergency population, compared to clinical or laboratory parameters alone. For the first time, the results of our study identified the combination of WBCs and negative urine test strip results as important predictors of AA diagnosis.

Similar to results of other studies, we found that symptoms of typical migratory pain and localized signs of peritonitis in the right lower quadrant are, although very specific, relatively rare in patients with AA (*7*). In those cases the diagnosis is not very difficult. However, a problem exists with all other cases where those specific symptoms are absent, and that is when the surgeon has to rely on some other parameters. In our study we found that no changes in appetite, diarrhea and dysuria are important negative predictors for AA, especially when combined with normal WBC and positive results of urine test strip analysis. However, the attending surgeons need always to bear in mind that there is no sign, symptom, or laboratory test, or their combination, that is 100% reliable in the diagnosis of acute appendicitis (*23*).

Although a bit peculiar, especially in the light of achieved high sensitivity (93.3%) in discriminating AA patients, and despite the literature findings that support the determination of CRP in the diagnosis of acute appendicitis, the logistic regression did not identify CRP concentration as a significant contributor to the acute appendicitis diagnosis in our study (*15*, *24*, *25*). Perhaps the underlying cause includes the same information that WBC and CRP offer, and thus one variable becomes redundant. Literature data even clearly demonstrates that CRP is not a good tool for helping the surgeon make the diagnosis of appendicitis and it should not be measured in suspected appendicitis, thus supporting our results (*26*, *27*). Regarding urine test strip analysis, the observed results are quite interesting. There were no differences in the proportion of patients with the positive urine test strip between the AA and the control group, however, the logistic regression analysis revealed the positive urine findings as a statistically significant negative predictor of AA. Logistic regression is a technique for analyzing problems in which there are one or more independent variables that determine an outcome. The goal of logistic regression is to find the best fitting (yet biologically reasonable) model to describe the relationship between the dichotomous characteristic of interest (dependent variable or outcome variable) and a set of independent (predictor) variables, and therefore yields completely reliable statistical results that are shown in this study (*28*). The literature regarding this specific scientific problem is rather scarce and very outdated, performed on very low number of patients, yielding questionable conclusions, and thus supporting the need for further investigations (*29*, *30*).

Mean platelet volume is proposed as a new potential biomarker for AA diagnosis, however our results have neither revealed any difference in MPV between the AA and control groups, nor has logistic regression analysis identified MPV as a significant variable in AA diagnosis (*23*).

The main limitation of our study is its case-control design and retrospective fashion. Nevertheless, the aim of this pilot study was only to identify potential candidates which could then be prospectively validated in another well designed study on a consecutive series of patients. Based on our findings we have already prepared such a protocol which has just recently been granted approval from the hospital Ethical Committee. However, to be accepted, any of the new method (set of parameters) should be as reliable as the old one. One of the prospective study goals will include calculating the Alvarado score and comparing the results with the proposed combination of signs and laboratory parameters that this paper identifies as most reliable. Hopefully, this study which is already under way will help confirm our findings and help improve the accuracy of AA diagnosis and decrease the rate of negative appendectomies. Until the final results of the prospective study, the surgical practice in our hospital will remain the same: the decision to operate will be made by senior attending surgeon. His decision will be based on clinical judgement and remain independent of modified diagnostic scoring system.

In conclusion, acute appendicitis in emergency setting may be successfully ruled in with high accuracy based on elevated WBCs and negative urine test strip in combination with signs of localized peritonitis, lack of appetite and absence of diarrhea. Considering broad differential diagnosis, especially in women, the positive urine test strip results enhances the rejection of the AA diagnosis. Since CRP did not contribute to the overall diagnostic accuracy, its use in AA diagnostic protocols is of no value. The key to successful diagnosis is responsible and thorough assessment, which contains adequate evaluation of laboratory parameters in combination with clinical exam.
